# Evidence of Physiological Decoupling from Grassland Ecosystem Drivers by an Encroaching Woody Shrub

**DOI:** 10.1371/journal.pone.0081630

**Published:** 2013-12-05

**Authors:** Jesse B. Nippert, Troy W. Ocheltree, Graciela L. Orozco, Zak Ratajczak, Bohua Ling, Adam M. Skibbe

**Affiliations:** 1 Division of Biology, Kansas State University, Manhattan, Kansas, United States of America; 2 Department of Forest and Rangeland Stewardship, Colorado State University, Fort Collins, Colorado, United States of America; 3 Department of Geography, Kansas State University, Manhattan, Kansas, United States of America; University of Illinois, United States of America

## Abstract

Shrub encroachment of grasslands is a transformative ecological process by which native woody species increase in cover and frequency and replace the herbaceous community. Mechanisms of encroachment are typically assessed using temporal data or experimental manipulations, with few large spatial assessments of shrub physiology. In a mesic grassland in North America, we measured inter- and intra-annual variability in leaf δ^13^C in *Cornus drummondii* across a grassland landscape with varying fire frequency, presence of large grazers and topographic variability. This assessment of changes in individual shrub physiology is the largest spatial and temporal assessment recorded to date. Despite a doubling of annual rainfall (in 2008 versus 2011), leaf δ^13^C was statistically similar among and within years from 2008-11 (range of −28 to −27‰). A topography*grazing interaction was present, with higher leaf δ^13^C in locations that typically have more bare soil and higher sensible heat in the growing season (upland topographic positions and grazed grasslands). Leaf δ^13^C from slopes varied among grazing contrasts, with upland and slope leaf δ^13^C more similar in ungrazed locations, while slopes and lowlands were more similar in grazed locations. In 2011, canopy greenness (normalized difference vegetation index – NDVI) was assessed at the centroid of individual shrubs using high-resolution hyperspectral imagery. Canopy greenness was highest mid-summer, likely reflecting temporal periods when C assimilation rates were highest. Similar to patterns seen in leaf δ^13^C, NDVI was highest in locations that typically experience lowest sensible heat (lowlands and ungrazed). The ability of *Cornus drummondii* to decouple leaf physiological responses from climate variability and fire frequency is a likely contributor to the increase in cover and frequency of this shrub species in mesic grassland and may be generalizable to other grasslands undergoing woody encroachment.

## Introduction

The worldwide encroachment of grassland systems by woody plants has been documented extensively [1], with specific examples from semi-arid [2]–[3], mesic [4]–[5], sub-arctic [6] and alpine grasslands [7], as well as savannas [8]–[10]. The often abrupt and non-linear transitions from grassland to woodland can typically be explained by positive feedbacks [11]–[15]. The diverse characteristics of ecosystems, attributes of encroaching species, rates of change, and local feedback dynamics have made the identification of general patterns difficult to isolate, resulting in many paradigms for shrub encroachment worldwide (e.g., elevated CO_2_, nutrient enrichment/depletion, ecohydrological change, altered disturbance regimes [11], [16]–[20]). Despite a similar theoretical trajectory of shrub encroachment into grasslands (e.g., positive feedbacks), we still lack commonalities in the local mechanism(s) facilitating woody encroachment across grasslands.

Within central North America, mesic grassland structure and function is commonly regulated by the interplay of multiple intermittent disturbances - fire, grazing, and climatic variability - which prevents the transition from grassland to woodland or forest [21]–[22]. These disturbances regulate ecosystem dynamics, maintaining grassland that is floristically diverse [23], productive [24], and capable of supporting large mammalian grazers [25]. Previous work has shown that the removal of fire from the system facilitates the rapid transition to a closed-canopy evergreen forest (*Juniperus virginiana*) within decades [5], [26], resulting in altered site biogeochemistry and reduced floristic diversity [26]–[28]. If fire frequency is changed (but not removed altogether), deep-rooted clonal shrubs (e.g., *Cornus drummondii, Rhus glabra*) increase in density and cover, with the most pronounced responses occurring in locations with soils greater than 0.5 m deep [4], [13]. Similar to the consequences of grassland conversion to an evergreen forest, conversion from mesic grassland to clonal shrubland alters ecosystem dynamics [5], occurs rapidly [13], [29], and results in semi-permanent ecosystem change (requiring mechanical removal and herbicide application for shrub removal [30]–[31]).

The clonal shrub rough-leaf dogwood (*Cornus drummondii*) is a grassland-encroaching species showing the greatest expansion in cover (from ∼0% to >30% cover) along the western edge of mesic prairie in central North America (Nebraska, Kansas, Oklahoma, USA). *C. drummondii* is a native species to this region, historically reported to occur in the transition zones and ecotones between C_4_-dominanted grasslands and riparian deciduous forests [32]. Previously, it has been shown that *C. drummondii* uses deeper soil-water sources compared to the coexisting C_4_ grass community [13], [33]–[34]. Using the stable isotopic signature of water from *C. drummondii*, a C_4_ grass (*Andropogon gerardii*), and soils at varying depths, Ratajczak et al. [13] showed that reliance on deep-water released *C. drummondii* from competition for water with the dominant grasses on site, which rely exclusively on surface soil moisture [35]. Use of water from deeper soil depths occurred in large shrub stems, as well as developing small stems, which lack a developed root profile [34], highlighting the rhizomatous-transfer of deep water among stems within the shrub island [13]. In locations with shallow soils (<50 cm depth), *C. drummondii* has patchy occurrence, does not increase in cover over time, and exhibits ∼50% mortality from a combination of water limitation and/or competition with grasses (Ratajczak unpublished). Thus, reliance on deeper soil moisture has the potential to minimize the negative consequences of short-term drought and high air temperature on the physiology and growth dynamics of *C. drummondii* (sensu: [36], [37]).

We used the stable carbon isotopic ratio (δ^13^C) of leaf tissue, which most broadly represents the integrated water-use efficiency (iWUE) and associated stomatal regulation during leaf development [38]–[39]. At each sample date, we revisited the same populations of clonal dogwood individuals (e.g., a ‘shrub island’). Using leaf δ^13^C we assessed leaf physiological responses over the broadest landscape gradients possible at this grassland site, with a monthly frequency during the summer from 2008–2011 (Table 1). We hypothesized that greater reliance on deeper soil-water by this woody shrub [13], [40] would minimize physiological variability to intra- and inter-annual climate variability because deep-water is less variable through time and typically more available than shallow soil moisture [40]. Access to water deep in the soil profile is not related to the treatment contrasts at this site (i.e., grazer presence, fire frequency) but does vary according to topography with uplands having shallower soils than lowlands. Thus, we also hypothesized that individual shrubs from locations with more bare soil and/or higher sensible heat (i.e., uplands, grazed locations, and recently burned locations) would have higher iWUE than shrubs from locations embedded in the herbaceous grass matrix with little bare soil and/or greater access to deep-soil water.

**Table 1:** Shrub island sample sizes among landscape contrasts.

		Fire Frequency (yr)					Topo. position		
		1	2	4	10	20	Upland	Slope	Lowland
Grazing	Yes	9	7	8	–	4	7	8	13
	No	17	5	15	4	7	10	10	28
Topo. position	Upland	4	1	7	2	3			
	Slope	5	4	6	–	3			
	Lowland	17	7	10	2	5			

76 total shrub islands were visited each sampling period.

## Materials and Methods

### Site Description

Research was conducted in mesic grassland at the Konza Prairie Biological Station (KPBS), located in the Flint Hills ecoregion of eastern Kansas, USA (39°05′ N, 96°35′ W). KPBS is a LTER (Long-Term Ecological Research) site dedicated to the study of tallgrass prairie ecology, jointly owned by the Nature Conservancy and Kansas State University. Permission to conduct this research and the associated research permit was approved by the KPBS director, Dr. John M. Briggs. KPBS is divided into 57 experimental landscape units (over 3487 ha) representing combinations of varying fire year return frequencies (1, 2, 4, 10, and 20) and grazing (bison, cattle, or no large mammalian grazers) treatments. Most prescribed fires occur in the spring (late March – early May) of each year, and have been imposed for the past 30+ years. The bison herd exists within the central 1012 ha of KPBS, maintained at a density of ∼300 adults since 1997, which results in a ∼25% removal of graminoid biomass [41]. Cattle occupy 844 ha on the eastern side of the site in cow-calf pairs from mid-May to September. No endangered or federally protected species were impacted by the research described here.

The site topography at KPBS is variable over small scales (∼0.1 km). Soil depth and structure varies with topography, as uplands have thin, rocky soil (Florence series) and lowlands have deeper silty-clay loams (Tully series). Changes in available soil moisture as a consequence of topographic position have been shown to result in lower aboveground net primary productivity in upland locations [24].

The regional climate for KPBS is characterized as mid-continental, with warm-wet summers, and cool-dry winters. Daily mean air temperature (1982-2011) varies between the coolest (−1.4±0.5°C - January) and warmest (26.1±0.3°C - July) months. From 1982–2011, mean annual rainfall (±1 SE) for KPBS was 805±35 mm, with ∼75% of the total amount occurring during the growing season (April–September).

### Sampling frequency/procedure

This research was conducted across 25 watersheds at KPBS, capturing the full suite of burn frequency by grazing by topography combinations at this site (Table 1). 76 *C. drummondii* islands—isolated clonal shrubs of many stems in a grassland matrix—were selected at random during the first sampling period in 2008, and then subsequently sampled for the duration of the project. Clonal shrub size was variable across the landscape, from ∼6–100 m^2^. The topography of the location for each sampled shrub was classified based on its relative position within each watershed (upland/ slope/lowland). Leaf samples were collected at approximate 4-week intervals during the growing season (May–September) from 2008–2010. In 2011, samples were collected in July and August only. Sample collection always occurred across the entirety of KPBS within a single day. Upon visiting each dogwood island, six emergent leaves were collected from the outer perimeter of each shrub island. Once collected, leaves were stored in coin envelopes and dried at 60°C for one week.

### Stable isotopic analysis

We measured the stable carbon isotope ratios and N concentrations of dried and ground leaf material from *C. drummondii* in the Stable Isotope Mass Spectrometry Laboratory (SIMSL) at Kansas State University. Samples were combusted with a CE1110 elemental analyzer (Carlo Erba Instruments, Milan, Italy) and coupled to a Delta Plus mass spectrometer (Thermo Electron Corporation, Bremen, Germany) for isotope analysis using a ConFlo II Universal Interface (Thermo Electron Corporation, Bremen, Germany). The isotopic ratio of samples was calculated using delta notation as:
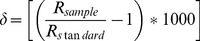
(1)where *R* is the ratio of the heavy to light isotope for the sample and standard, respectively. The within-run variability estimated as the SD of working standards was always <0.05‰, and the between-run variability, estimated as the difference between the measured value of a working standard and its calibrated value, was always <0.05‰.

Integrated Water-Use Efficiency (iWUE) is related to δ^13^C of plant tissue through the ratio of internal to atmospheric CO_2_ concentration (c_i_/c_a_). iWUE is a measure of the amount of carbon fixed during photosynthesis (A) relative to stomatal conductance (g) and can be estimated from c_i_/c_a_:
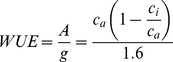
(2)


The ^13^C of plant tissue is determined by the ^13^C of the CO_2_ source and c_i_/c_a_, and is expressed as: 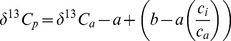
(3)


Where δ^13^C _p_ and δ^13^C_a_ are the carbon isotope ratios of the plant tissue and atmospheric air, respectively. ‘a’ and ‘b’ are discrimination factors due to diffusion through the stomata and carboxylation, which are relatively constant values of 4.4‰ and 29‰ [38]. c_i_/c_a_ responds to changes in photosynthetic rate and stomatal conductance, just as iWUE, and can be solved using eq. 3. When using δ^13^C as a proxy for iWUE, smaller values indicate lower iWUE.

### NDVI calculation

Using ArcGIS 10.0 Spatial Analyst Tools (ESRI 2012) we extracted values from a calculated NDVI (normalized difference vegetation index) product derived from four hyperspectral flights of KPBS in 2011. These hyperspectral data were collected by an aircraft mounted AISA camera and atmospherically corrected with FLAASH resulting in a 2 m×2 m resolution data product. NDVI was calculated as:
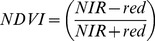
(4)


Due to variations in spectral resolution over the season in 2011, the values used for red (620–750 nm) and NIR (750–1400 nm) varied by date (Red = R684.02 nm, NIR = R871.17 nm, for May–August, and Red = R685.2 nm, NIR = R875.96 nm, for September). These values were chosen to represent each red and NIR spectrum edge respectively. NDVI was calculated at each shrub island centroid (4 m^2^) for each of the same 76 individuals previously measured for leaf δ^13^C analysis. Because all shrubs were larger than the resolution of the imagery collected, no herbaceous vegetation from the circumference of the shrub was included in the NDVI data, and little to no herbaceous vegetation existed under the shrub canopy [13].

### Statistical Analysis

All statistical analyses were performed using the open-source R statistical software package [42]. Climate data from the KPBS headquarters were harvested from the ClimDB database (http://www.fsl.orst.edu/climhy/) and were summarized for growing season statistics only, which was defined as DOY 105–258 (April 15- September 15). Stable isotope and N content data were verified for normality, and any outliers (defined as 2 standard deviations beyond the overall mean) were removed from the data set. Linear mixed-effects models were used to analyze the data using the ‘nlme’ package in R [43]. Linear mixed-effects models accommodate unbalanced and grouped data by associating the random effects of individual experimental units to the observations of interest [44]. For the leaf δ^13^C data, fixed-effects included all spatial contrasts- grazing (grazed/ungrazed), topographic position (upland, slope, lowland), fire frequency (1, 2, 4, 10, 20 years), as well as temporal contrasts - year and day of year sampled. Because day of year is nested within year, this interaction term was not included as a fixed effect. In the random effects structure of the model, watershed (location of shrub sampled) within DOY (sampling day) was used. For the NDVI data, fixed-effects included grazing, fire, topography and day of year and their interactions, and watershed was a random effect. To assess the relationship between leaf δ^13^C and %N_(mass)_, a simple linear regression was fit to all data from 2008–2011.

## Results

**Figure 1:**
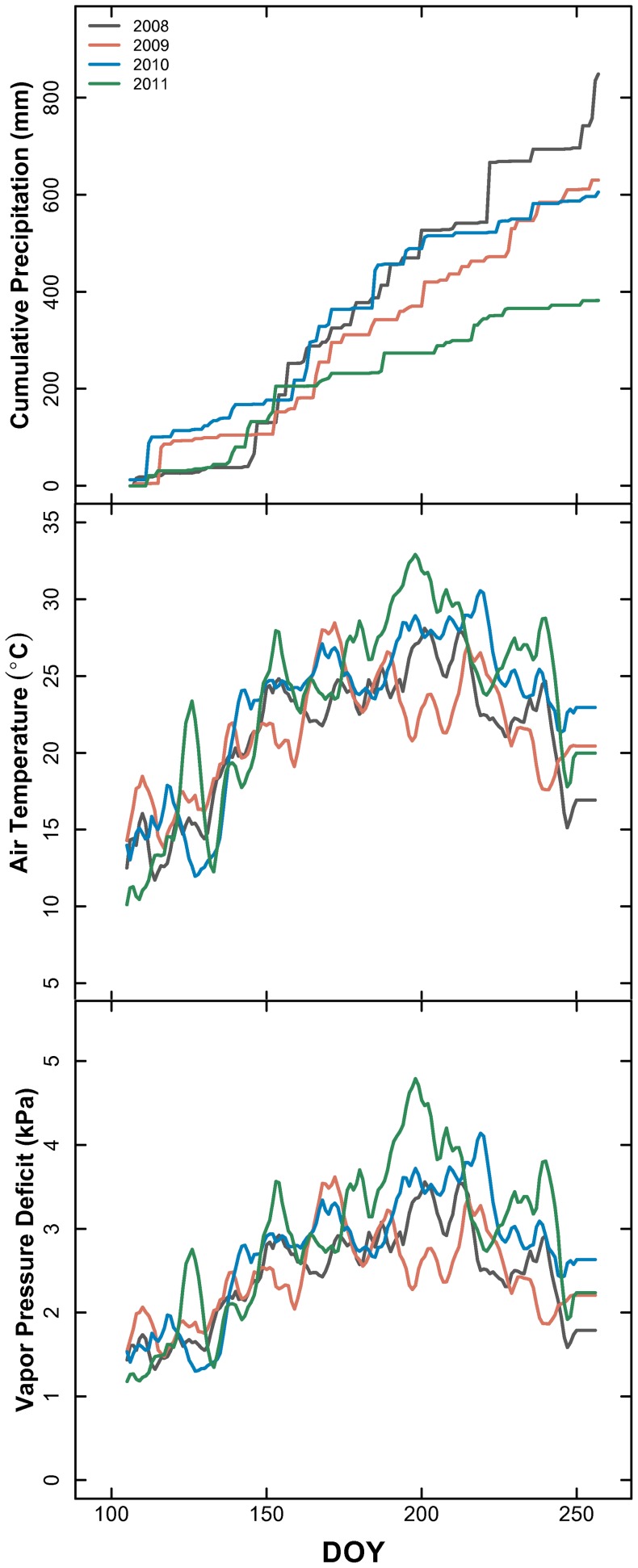
Konza climate from DOY 105–259 for the four years of this study: a – cumulative precipitation (mm); b – mean daily air temperature (°C); c – mean daily vapor pressure deficit (kPa).

Cumulative growing season precipitation (from DOY 105–258) averaged 520.3 mm from 1982–2011, but differed between the 4 years studied (Fig. 1a). The amount of growing season precipitation was above the long-term average (520.31 mm from 1982–2011) during 2008–2010. Precipitation in 2008 was the greatest for the 4 years of this study (848.6 mm), followed by 2009 (630.0 mm) and 2010 (647.6 mm), although the temporal pattern of precipitation and corresponding intra-annual variability differed among these two years (Fig. 1a). The amount of precipitation received in 2011 was the lowest of the 4 years (406.1 mm). The long-term average for growing season temperature from 1982–2011 was 22.05°C. 2008 & 2009 were cooler than average, and 2011 was 1.1°C higher than average during the growing season. 2011 was 2.83°C warmer than the next warmest year (2010) from DOY 191–211 (Fig. 1b). During this same period of time, VPD during 2011 was on average 0.72 kPa greater than 2010 (Fig. 1c).

**Figure 2:**
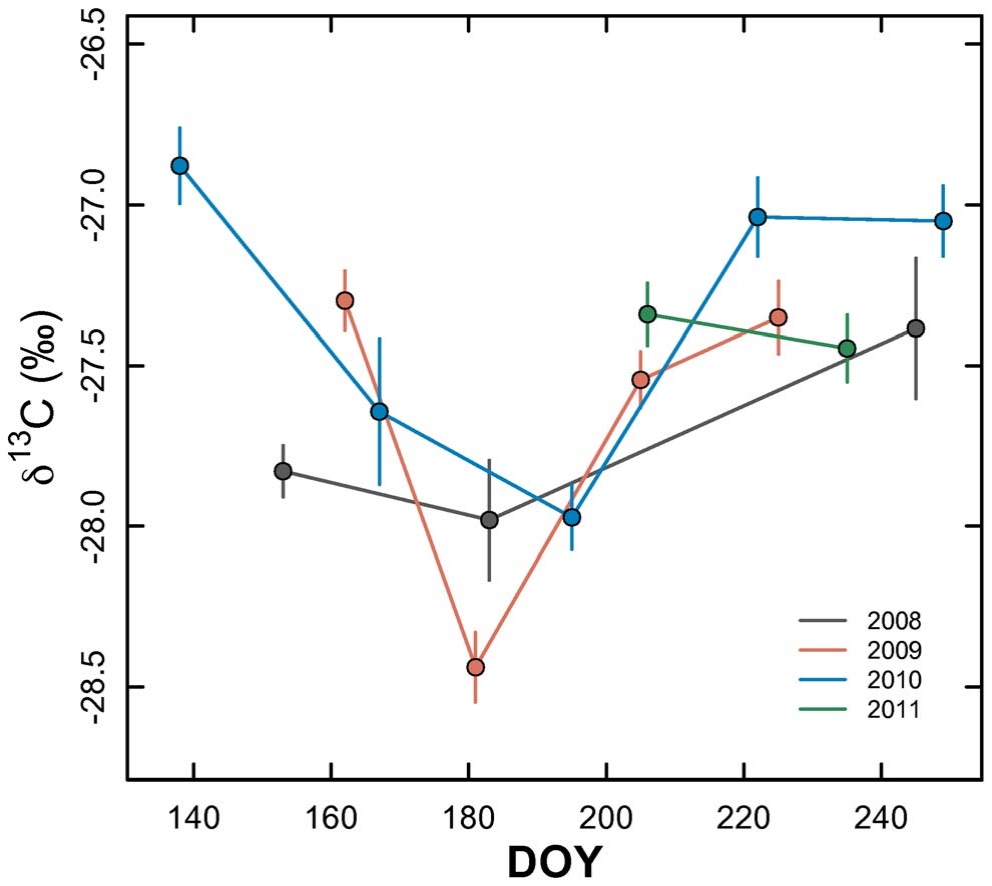
Changes in mean leaf δ^13^C (‰) (±1SE) by day of year across the growing season from 2008–2011.

**Figure 3:**
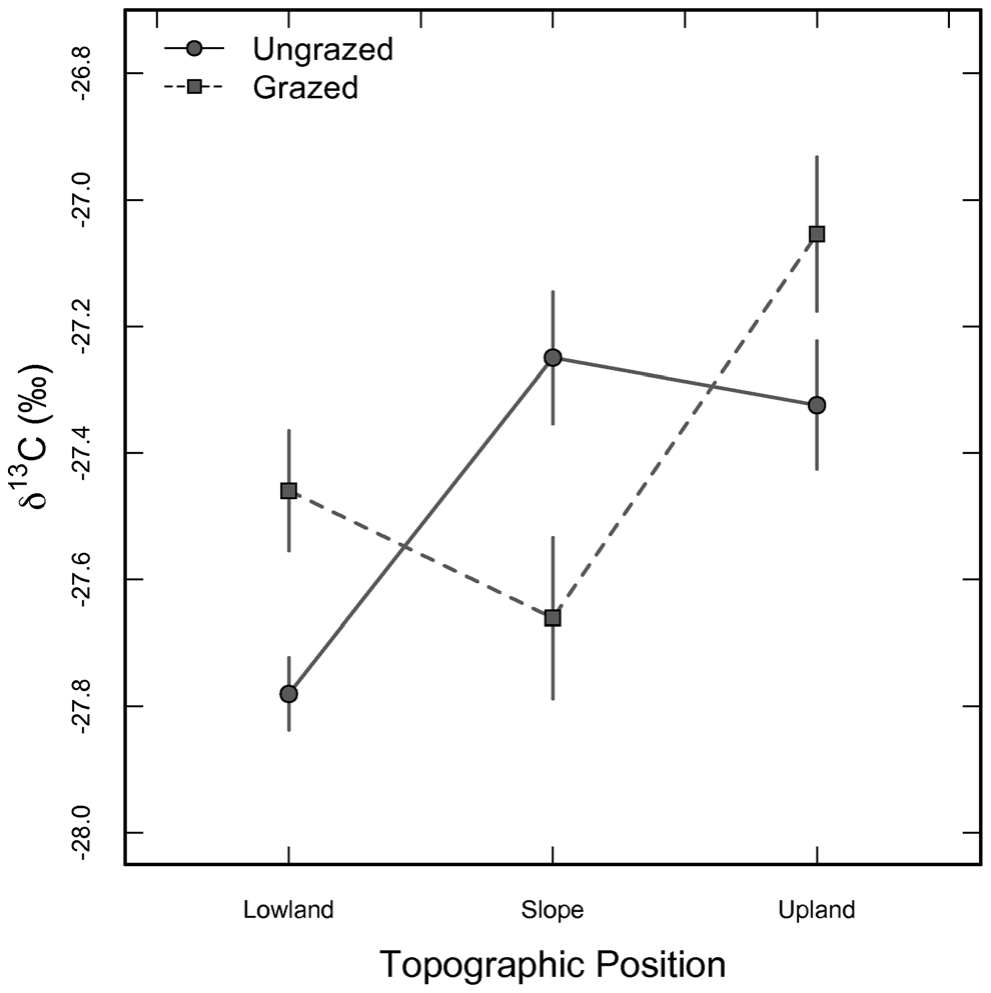
Changes in leaf δ^13^C (‰) by topographic positions (upland, slope, lowland) and according to grazing contrasts (grazed, ungrazed). Data are mean values (±1SE) from 2008–2011.

Leaf δ^13^C in *C. drummondii* did not vary among years (p = 0.287, F = 2.07) or days sampled within years (p = 0.512, F = 0.47) (Table 2, Fig. 2), despite the varying climate histories from 2008–2011 (Fig. 1). A significant grazing by topographic position interaction was present (p<0.001) for leaf δ^13^C (Table 2, Fig. 3), with leaves from slope positions in grazed watersheds having smaller δ^13^C values than leaves from ungrazed slope positions. When assessed by topographic position on the landscape, leaves from *C. drummondii* in upland locations had larger δ^13^C values (i.e. more enriched in ^13^C) compared to individuals from topographic slopes and lowland positions, respectively (Fig. 3). A similar difference in leaf δ^13^C existed in leaves collected from grazed locations, with leaves from grazed sites tending to be more enriched in ^13^C than ungrazed sites (Fig. 3). Leaf δ^13^C did not vary (p>0.05) according to burn frequency at KPBS for the years studied (Table 2). A significant, but weak, positive correlation was present (p = 0.001; r^2^ = 0.01; y = 0.26x−27.99) between leaf δ^13^C and %N using data from all years combined (data not shown).

**Table 2:** Mixed-effects model results for leaf δ^13^C (‰) among landscape contrasts including: grazing (grazed/ungrazed), topographic position (upland, slope, lowland), and burn frequency (1, 2, 4, 10, 20) and temporal contrasts (year and day of year).

	numDF	denDF	F-value	p-value
Grazing	1	682	3.85	0.05
Position (topo)	2	682	12.81	<.0001
Burn_freq	1	682	3.37	0.0669
Year	1	2	2.07	0.2872
DOY	1	9	0.47	0.5118
G:P	2	682	7.7	0.0005
G:Bf	1	682	1.58	0.2087
P:Bf	2	682	2.26	0.105
G:Year	1	682	0.58	0.4471
G:DOY	1	682	1.18	0.2777
P:Year	2	682	0.39	0.6768
P:DOY	2	682	0.97	0.3783
Bf:Year	1	682	0.82	0.3648
Bf:DOY	1	682	2.05	0.1531
G:P:Bf	2	682	0.7	0.4962
G:P:Year	2	682	0.03	0.9666
G:P:DOY	2	682	0.06	0.9449
G:Bf:Year	1	682	1.7	0.1931
G:Bf:DOY	1	682	0.63	0.4279
P:Bf:Year	2	682	0.36	0.6986
P:Bf:DOY	2	682	1.08	0.3395
G:P:Bf:Year	2	682	1.07	0.3443
G:P:Bf:DOY	2	682	0.57	0.5677

Random effects include the watershed within day sampled.

**Figure 4:**
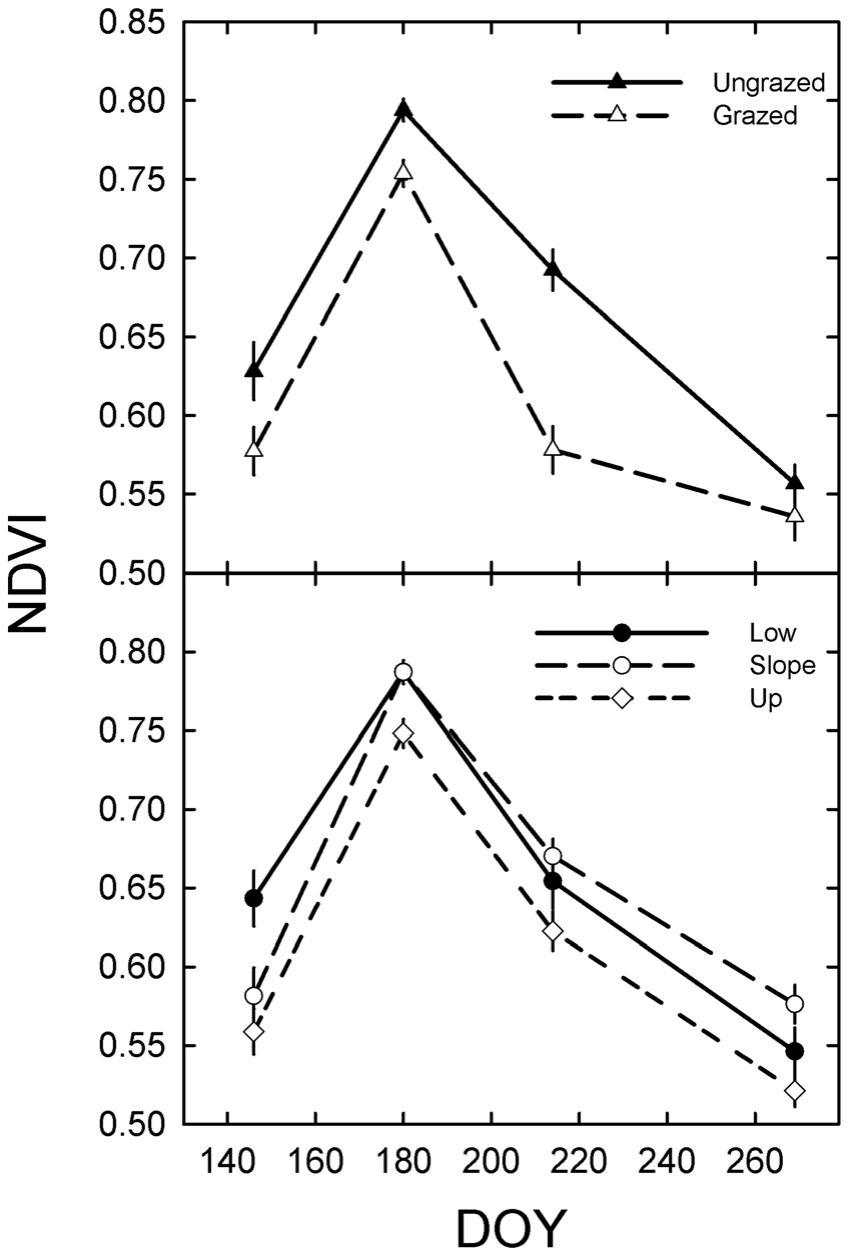
Changes in mean NDVI (±1SE) by day of year in 2011. Each estimate of NDVI represents the image captured at ∼2×2 m resolution for each individual dogwood island on the landscape. Panel ‘a’ shows differences according to topographic positions, while panel ‘b’ shows differences by grazing contrasts.

NDVI from each shrub island varied significantly (p<0.001) among the 4 time periods measured in 2011 (Table 3) with a parabolic shape, with peak values near DOY 180 (Fig. 4). No differences were present based on burn frequency across the landscape, but NDVI was significantly lower in grazed areas (Fig. 4a) and upland topographic positions (Fig. 4b).

**Table 3:** Mixed-effects model results for NDVI by day of year and by landscape contrasts: grazing (grazed/ungrazed), topographic position (upland, slope, lowland), and burn frequency (1, 2, 4, 10, 20).

	numDF	denDF	F-value	p-value
Grazing	1	238	6.968	0.0088
Position (topo)	2	238	3.2293	0.0413
Burn_freq	1	238	0.3121	0.5769
DOY	1	238	22.1902	<.0001
G:P	2	238	3.7687	0.0245
G:B	1	238	0.0334	0.8551
P:B	2	238	0.1706	0.8432
G:DOY	1	238	0.0711	0.7899
P:DOY	2	238	1.3037	0.2735
B:DOY	1	238	0.01	0.9203
G:B:P	2	238	1.2241	0.2959
G:P:DOY	2	238	0.4158	0.6603
G:B:DOY	1	238	0.6971	0.4046
DOY:B	2	238	0.2106	0.8103
4-way	2	238	0.0732	0.9295

Random effects include the watershed sampled in 2011.

## Discussion

Measuring the leaf δ^13^C across 4 growing seasons and a broad spatial template allowed us to assess the integrated physiological dynamics for *C. drummondii*, the predominate woody encroacher for this mesic grassland. Compared across years, patterns in leaf δ^13^C were similar (Fig. 2), despite a doubling in inter-annual precipitation and variable air temperature and vapor pressure deficits (Fig. 1). The similarity in leaf δ^13^C across years suggests that integrated leaf physiological function was insensitive to ambient annual climate dynamics. This result directly contrasts previous studies on the herbaceous species at KPBS, which have shown this grassland community to be very responsive to intra- and inter-annual variability in climate [24], [45]–[46].

Leaf δ^13^C did not vary intra-annually (Table 2), but leaf δ^13^C values were highest early the growing season, lowest mid-summer (early July), and then increased to higher values later in the growing season (Fig. 2). Using small plots, McCarron and Knapp [33] measured instantaneous water-use efficiency for *C. drummondii*, and reported non-significant seasonal changes (but a trend with lowest WUE mid-season and highest WUE late in the growing season). Similar subtle responses for instantaneous WUE across the growing season despite changes in surface soil moisture have been shown for other grassland-encroaching woody species, including *Prosopis glandulosa* [47], and *Juniperus virginiana* [48] and for shrubs coexisting in grasslands (*Amorpha canescens* – [49]; *Artemisia cana* and *Rhus trilobata* – [50]). The seasonal pattern in leaf δ^13^C here (Fig. 2) may reflect contributions of stored carbohydrates in the production of new leaf biomass early in the growing season. In this scenario, the first leaves produced utilize a greater proportion of stored carbohydrates, with an isotopic signature reflecting carbon fixed at the end of the previous growing season. This leaf δ^13^C would vary from carbon fixed mid-summer when leaves are at peak photosynthetic activity [51] and leaf δ^13^C would likely reflect recently fixed carbon. Remobilization of stored carbohydrates into developing leaves has been shown previously using δ^13^C in shrub species [52]. NDVI calculated for individual shrub islands in 2011 had a significant seasonal trend (Table 3), with a maximum ‘canopy greenness’ in late June and subsequent declines over the remainder of the season (Fig. 4). The period with the highest NDVI here corresponds with previous work showing the highest photosynthetic rates for *C. drummondii* mid-summer [51].

Previously, a shrub δ^13^C isoscape was conducted along a topographic gradient in subtropical Texas savanna [53]. Bai et al. [53] reported a ∼1.5‰ range in δ^13^C across landscape and temporal contrasts, with the greatest differences between species and among contrasts of topographic positions. In our results here from 2008–11, the site-wide seasonal range of was also narrow (∼1.5‰) despite individuals being measured across the full suite of site treatments (topography, fire, grazing) (Table 1). By comparison, a site-wide sampling of 310 separate C_3_ species was conducted on Konza Prairie in 2009–10 across similar contrasts in landscape gradients [54]. In the supplemental data of Craine et al. [54], leaf δ^13^C varied from −35 to −25‰ (mean = −29.7‰, SD = 1.5‰) illustrating the role of species differences, seasonal and landscape variability as a driver of leaf δ^13^C at this site. It was previously shown that leaf %N accounted for some of the topographic variability in leaf δ^13^C for subtropical woody shrubs [53], [55]. In this study, a significant statistical relationship between %N and leaf δ^13^C was present (p = 0.001), but explained very little of the overall variability (r^2^ = 0.01).

We hypothesized that the use of deep soil-water by *C. drummondii* would minimize variability in leaf δ^13^C across years with varying rainfall patterns (and microclimates) and among fire, grazing, and topographic contrasts, because a reliable water source may minimize the negative physiological effects of short-term drought, high temperature, and high evaporative demand (i.e. high VPD). As noted previously, leaf δ^13^C did not vary according to sample date or year, but a topography*grazing interaction exists (Fig. 3). *C. drummondii* from lowlands regions (with deeper soils) had smaller leaf δ^13^C (and lower iWUE) than individuals from uplands, which typically have shallower, coarse soils and reduced soil moisture [24]. Leaf δ^13^C was typically larger in grazed versus ungrazed locations, suggesting higher iWUE for shrubs in grazed locations. On Konza Prairie, ‘slope’ positions typically represent mid-points between thin-soil uplands and deep-soil lowlands. Thus, soil-depth on slopes is variable site-wide. Here, leaf δ^13^C on slopes was higher in ungrazed versus grazed locations (Fig. 3), likely reflecting differences in soil depth among slopes rather than specific impacts of grazing at these locations. Uplands and grazed locations typically have less aboveground herbaceous biomass and greater occurrence of bare soil, resulting in higher sensible heat fluxes compared to locations with greater herbaceous biomass surrounding each individual shrub island. It is likely that higher iWUE reflects physiological responses to higher sensible heat at upland and grazed locations. Similar to leaf δ^13^C, NDVI for *C. drummondii* varied according to topographic position and by grazing contrasts (Fig. 4) with the highest canopy greenness in ungrazed and lowland topographic positions.

Reliance on a deeper water source provides a physiological advantage for woody plants by minimizing the impacts of within-season drought and allowing for longer periods of growth than the herbaceous community [47], [50]. In addition, the clonal redistribution of water via rhizomes supports the production of new stems and the expansion of shrub islands across the landscape independent of short-term variability in climate [13], [34]. Indeed, with access to deep-soil water a hysteresis loop may exist in this system because the severity of drought that governs seedling establishment is expected to be lower than that required to cause adult shrub mortality. Woody shrubs without access to deep-water sources (existing in locations with shallow soils) have greater susceptibility to drought and stress-induced physiological adjustment [56]–[57].

The spatial and temporal partitioning of water resources between coexisting woody plants and grasses has been previously shown in a variety of North American grasslands [35], [58]–[59]. In karst landscapes of the Edwards Plateau, TX, an extended drought (and reduced availability of deep soil water) lowered carbon uptake and resulted in physiological stress by woody plants in a savanna landscape [57]. At Konza Prairie, a drought of sufficient magnitude to lower water availability at soil depths below 1 m are unknown, as are the potential physiological and stress-responses by woody shrubs reliant on these deeper water sources. If future climate changes alter seasonal rainfall distribution resulting in more rainfall events and amounts of rainfall in the non-growing season (winter), greater infiltration to deeper soil depths is likely to occur. In this scenario with greater soil moisture at deeper soil depths, the shrub communities may be buffered from future summer-time droughts.

In combination with physiological studies from other sites [47]–[48], [50], [53], the results of this study suggest that deep-rooted woody species like *C. drummondii* may have growth and cover dynamics that are decoupled from several of the local environmental drivers that govern the dynamics of the herbaceous grassland communities (here, climate variability and fire). Bai et al. [53] concluded that the greater reliance on deeper soil-water sources may allow shrub species to be decoupled from microclimate and temporal variability in near-surface soil moisture. This decoupling from key ecosystem drivers results in a fundamentally different response for the woody encroaching species compared to the herbaceous grassland community, allowing the woody species to avoid competition for water in both time and space. Thus, in addition to the large-scale forcing [6], [18] and land-use changes [8], [17], rapid rates of woody encroachment in some regions may occur because post-establishment encroaching species become decoupled from the typical system dynamics that regulate the dominant species of the ecosystem.
